# Drug use disorder and risk of incident and fatal breast cancer: a nationwide epidemiological study

**DOI:** 10.1007/s10549-020-05998-4

**Published:** 2020-11-06

**Authors:** Disa Dahlman, Hedvig Magnusson, Xinjun Li, Jan Sundquist, Kristina Sundquist

**Affiliations:** 1 Center for Primary Health Care Research, Department of Clinical Sciences, Lund University/Region Skåne, Box 503 22, Malmö, Sweden; 2 Department of Family Medicine and Community Health, Icahn School of Medicine At Mount Sinai, New York, USA; 3 Center for Community-Based Healthcare Research and Education (CoHRE), School of Medicine, Shimane University, Matsue, Japan

**Keywords:** Breast cancer, Drug abuse, Mortality, Sweden

## Abstract

**Purpose:**

Breast cancer is one of the most common cancer forms in women and it is often detected by screening. However, women with drug use disorders (DUD) are less likely to be reached by screening programs. In this study, we aimed to investigate breast cancer incidence, mortality and stage at time of diagnosis among women with DUD compared to the general female population in Sweden.

**Methods:**

We performed a follow-up study based on Swedish national register data for the period January 1997–December 2015. The study was based on 3,838,248 women aged 15–75 years, of whom 50,858 were registered with DUD. Adjusted hazard ratios (HRs) for incident and fatal breast cancer, and cancer stage at time of diagnosis, were calculated for women with and without DUD using Cox regression analysis.

**Results:**

DUD was associated with incident breast cancer (HR 1.08, 95% confidence interval [CI] 1.02–1.14, *p* = 0.0069), fatal breast cancer (HR 1.60, 95% CI 1.42–1.82, *p* < 0.001), and stage IV breast cancer, i.e. metastasis at diagnosis (HR 2.06, 95% CI 1.44–2.95, *p* < 0.001).

**Conclusions:**

Women with DUD were identified as a risk group for incident, fatal and metastasized breast cancer, which calls for attention from clinicians and policy makers. Cancer screening attendance and other healthcare seeking barriers are likely to affect the risk increase among women who use drugs; however, more research is needed on the underlying mechanisms.

**Electronic supplementary material:**

The online version of this article (10.1007/s10549-020-05998-4) contains supplementary material, which is available to authorized users.

## Introduction

Drug use disorders (DUD) are associated with a range of adverse health outcomes including increased mortality rates [1, 2]. For example, patients in opioid substitution treatment (OST) are shown to have significantly higher non-drug-related mortality, including in cancer, than the general population [3, 4]. The mortality rates are especially increased in patients older than 55 years [3], which is worrying since the OST populations are aging [5]. Psychiatric comorbidity is also common among people who use drugs and people in OST [6–8].

The adverse health outcomes associated with DUD could partly be related to unmet health care needs [9–11] including non-participation in cancer screening programs, such as for breast cancer [12, 13], cervical cancer [13–15] and colorectal cancer [13]. It is therefore likely that women with DUD have higher mortality in common cancers, e.g. breast cancer, and a delayed diagnosis.

In addition, women with DUD often have poor socioeconomic status (SES), which may further deteriorate their chances of achieving good health. While high SES is associated with increased incidence of breast cancer, breast cancer survival is lower among women with low SES [16], which in turn is related to lifestyle factors. For example, excessive alcohol consumption is associated with breast cancer [17, 18], and is also overrepresented among people with DUD [11, 19, 20]. Tobacco smoking is also associated with low SES [21] and has been identified as a risk factor for breast cancer [22]. In addition, tobacco smoking is highly prevalent among people with DUD [19, 23, 24].

Breast cancer is the most commonly diagnosed cancer and the leading cause of cancer death among women globally, with over two million women being diagnosed each year [25]. In Sweden, breast cancer is one of the leading causes of death among middle aged women [26]. Breast cancer screening through mammography every 18–24 months is therefore offered to Swedish women at no or a low cost between 40 and 74 years of age [26], since 1997. The screening program reduces the breast cancer mortality with 16–25% [26]. All healthcare in Sweden is tax financed and strongly subsidized for the individual [27].

Despite the critical need of more knowledge on whether women with DUD have a higher breast cancer incidence or a poorer prognosis when diagnosed with breast cancer, we have only found one previous study on DUD and breast cancer [4]. In this study, opioid dependence was associated with lower breast cancer mortality compared to the general female population in Australia [4]. We therefore aimed to examine breast cancer incidence, mortality, and stage at the time of diagnosis among women with DUD compared to the general female population in Sweden, after taking potential confounders, including SES, into account.

## Methods

### Data sources

We performed a retrospective cohort study of national register data. A dataset was constructed through linkage of data from the following Swedish national registers: the Swedish National Patient Register (NPR) for inpatient care (1964–2015) and outpatient care (2001–2015), the Total Population Register, the Swedish Cause of Death Register (1961–2015), the Swedish Prescribed Drug Register (2005–2015), the Swedish Cancer Register (1958–2015), the Crime Register (1973–2015), and the Suspicion Register (1998–2015). All linkages were performed using the national 10-digit civic registration number, which is assigned to each person in Sweden upon birth or immigration to the country. This number was replaced by a serial number to ensure the integrity of all individuals. The registers contain, e.g., individual-level data on age, sex, education, hospital admissions, dispensed drugs and breast cancer diagnoses on a nationwide basis, including the study population of women aged 15–75 years in Sweden.

Ethical approval for the study was obtained from the Regional Ethical Review Board in Lund (file number 2012/795).

### Participants

Women 15–75 years of age at January 1, 1997 were included in the study. We excluded individuals with a diagnosis of breast cancer between January 1, 1991 and December 31, 1996, i.e., those with an earlier diagnosed cancer (*n* = 37,533). Finally, a total of 3,838,248 individuals were included in the study (Supplementary Fig. 1). The comparison group were women without an incident of DUD.

### Exposure

*Drug use disorders (DUD)* were identified in the following registries any time during the study period: the NPR by relevant ICD-10 codes (F10–F19: mental and behavioral disorders due to psychoactive substance use, except those due to alcohol or tobacco); the Suspicion Register, which records suspected crimes related to drug use by codes 3070 (driving under the influence of drugs), 5010 (drug possession), 5011 (drug use), and 5012 (drug possession and use); and the Crime Register, which records convictions by references to laws covering narcotics (law 1968:64, paragraph 1, point 6) and drug-related driving offenses (law 1951:649, paragraph 4, subsection 2 and paragraph 4A, subsection 2). DUD was also identified in individuals (excluding any other cancer patients) in the Prescribed Drug Register who had filled prescriptions for hypnotics and sedatives (Anatomical Therapeutic Chemical [ATC] Classification System N05C and N05BA) or opioids (ATC: N02A) in average dosages of more than four defined daily doses a day for 12 months.

### Outcome variables

#### Incident breast cancer

The outcome variable was a diagnosis of breast cancer in the Swedish Cancer Register during the study period (1997–2015). The 7th revisions of the International Classification of Diseases (ICD-7) was used to identify breast cancer (ICD-7 170).

#### Fatal breast cancer

The outcome variable was a diagnosis of breast cancer in the Swedish Cause of Death Register during the study period, using the 10th revision of the ICD to identify breast cancer (ICD-10 C50).

#### Breast cancer stage at time of diagnosis

Stage 0–IV, obtained from the Swedish Cancer Register, based on the TNM classification. TNM is short for Tumor (tumor characteristics, including size), Nodes (spread to nearby lymph nodes), and Metastases (spread to other parts of the body).

### Covariates

*Age* was recorded at January 1, 1997 into three groups: 15–34 years, 35–49 years and 50–75 years.

*Educational attainment* as of January 1, 1997 was categorized as ≤ 9 years (partial or complete compulsory schooling), 10–12 years (partial or complete secondary schooling) and > 12 years (some or completed college and/or university studies). Prior to the statistical analysis, educational attainment was dichotomized into “up to 12 years” and “ > 12 years”.

*Marital status* as of January 1, 1997 was classified as married/cohabiting, unmarried, divorced or widowed. We used two categories in the analysis, i.e., married/cohabiting vs. not married/cohabiting (including all unmarried, divorced and widowed women).

*Region of residence* was recoded into “large city” and “small city/countryside” from the original categories “large city”, “southern Sweden” and “northern Sweden”. Large cities were defined as one of the three largest cities in Sweden (Stockholm, Gothenburg and Malmö). “Southern Sweden” and “northern Sweden” were recoded into “small city/countryside”.

*Social welfare* was defined as Yes (received) and No (not received).

*Alcohol use disorder* was identified according to ICD-10 F10 and K70 during the study period.

### Statistical analysis

Cox regression models were used to estimate Hazard ratios (HRs) with a 95% confidence interval (CI) to test for the association between DUD and incident breast cancer, fatal breast cancer, and cancer stage at diagnosis in the women. The women were followed for time to the first breast cancer outcome, death, migration from Sweden, or until December 31, 2015. The follow-up started on January 1, 1997 and ended on December 31, 2015. The average follow-up time was 16.6 ± 3.6 years. We used two models: Model 1 was adjusted for age at entering the cohort; Model 2 was adjusted for age and the covariates listed above. The proportionality assumptions were checked by plotting the incidence rates over time and by calculating Schoenfeld (partial) residuals and these assumptions were fulfilled (Supplementary Fig. 2).

All statistical analyses were performed using SAS version 9.4 (SAS Institute Inc. Cary, NC, USA). A two-tailed *p*-value of < 0.05 was used for statistical significance for the outcomes.

A sensitivity analysis was conducted using data obtained from the Swedish Medical Birth Register. Information on the smoking history, parity, age at first delivery and body mass index (BMI) of the women (mothers) from the Medical Birth Register was identified and included in the analysis. The initial population for the women was 1,349,971 because data on smoking were only collected in the latter age cohorts of pregnant women. Another sensitivity analysis was conducted using data obtained from the Swedish Multi-generation Register. Information on the family history (mothers or sisters) of breast cancer was identified. The initial population for the women was 2,458,279 because data on the second-generation daughters were collected only among those born in 1932 and onwards due to the nature of the register. An additional analysis was performed with the exposure variable DUD replaced with opioid use disorder, defined as ICD-10 codes F11, and retrieved from the NPR.

## Results

### Baseline characteristics

The study population included 3,838,248 women aged 15–75 years in January 1, 1997. The mean age was 42.5 years (standard deviation 16.9), 38.7% were married/cohabiting, 41.6% were living in small cities/countryside, 44.1% had more than 12 years of education, and 7% had received social welfare (Table 1). DUD was incident in 1.3% of the study population (*n* = 50,858). Women with DUD were younger than the comparison group, had shorter education, had more often received social welfare, were more often living in large cities, and were more often not married/cohabiting.

**Table 1:** Population and number of events of incident and fatal breast cancer

	Total population		Incident breast cancer		Fatal breast cancer	
	No	%	No	%	No	%
Drug use disorders						
Non	3,787,390	98.7	107,676	98.8	16,692	98.4
Yes	50,858	1.3	1310	1.2	267	1.6
Age groups (years)						
15–34	1,443,369	37.6	11,132	10.2	1061	6.3
35–49	1,013,516	26.4	32,798	30.1	3868	22.8
50–75	1,381,363	36.0	65,056	59.7	12,030	70.9
Educational attainment						
< 12 years	1,732,700	45.1	64,636	59.3	11,324	66.8
12 + years	1,694,316	44.1	43,300	39.7	5307	31.3
Unknown	411,232	10.7	1050	1.0	328	1.9
Social welfare						
Non	3,569,683	93.0	103,761	95.2	16,124	95.1
Yes	268,565	7.0	5225	4.8	835	4.9
Region of residence						
Large city	1,594,505	41.5	54,934	50.4	8432	49.7
Small city/countryside	1,597,877	41.6	51,638	47.4	7839	46.2
Unknown	645,866	16.8	2414	2.2	688	4.1
Marital status						
Married/cohabiting	1,484,886	38.7	62,933	57.7	9097	53.6
Not married/cohabiting	1,707,496	44.5	43,639	40.0	7174	42.3
Unknown	645,866	16.8	2414	2.2	688	4.1
Alcohol use disorder						
Non	3,775,382	98.4	107,101	98.3	16,777	98.9
Yes	62,866	1.6	1885	1.7	182	1.1
All	3,838,248	100.0	108,986	100.0	16,959	100.0

### Incident breast cancer and DUD

In the study population, 2.8% (*n* = 108,986) received a breast cancer diagnosis. Cox regression analysis adjusted only for age (Model 1) showed a statistically significant positive association between DUD and breast cancer (HR 1.14, 95% CI 1.08–1.20; Table 2). The association remained in Model 2 after adjustment also for educational attainment, social welfare, region of residence, marital status and alcohol use disorder (HR 1.08, 95% CI 1.02–1.14). Inverse associations were found between breast cancer and education < 12 years (HR 0.77, 95% CI 0.76–0.78) as well as between breast cancer and social welfare (HR 0.89, 95% CI 0.87–0.92). Living in a large city (HR 1.36, 95% CI 1.34–1.37), being not married/cohabiting (HR 1.14, 95% CI 1.12–1.15), of age 35–49 (HR 4.58, 95% CI 4.48–4.68) and 50–75 years (HR 7.96, 95% CI 7.79–8.13), and alcohol use disorder (HR 1.11, 95% 1.06–1.17) were all positively associated with incident breast cancer in Model 2.

**Table 2:** Association of drug use disorders (DUD) and incident breast cancer

Covariates	Person years follow-up	Model 1				Model 2			
		HR	95% CI		*p*-value	HR	95% CI		*p*-value
DUD (vs. non)	852,294	1.14	1.08	1.20	< 0.001	1.08	1.02	1.14	0.0069
Age (vs. age 15–34 years)									
35–49	17,472,852	4.20	4.12	4.30	< .0001	4.58	4.48	4.68	< .0001
50–75	21,504,144	6.86	6.72	7.00	< .0001	7.96	7.79	8.13	< .0001
Educational attainment < 12 years (vs. 12 + years)	34,957,420					0.77	0.76	0.78	< .0001
Social welfare (vs. no social welfare)	4,504,849					0.89	0.87	0.92	< .0001
Region of residence large city (vs. small city/countryside)	26,375,130					1.36	1.34	1.37	< .0001
Marital status not married/cohabiting (vs. married/cohabiting)	28,414,388					1.14	1.12	1.15	< .0001
Alcohol use disorder (vs. non)	1,025,119					1.11	1.06	1.17	< .0001

*N* = 3,838,248. Total person years follow-up 63,903,192

*HR* hazard ratio, *CI* confidence interval

Model 1: Adjusted for age; Model 2: Adjusted for age, educational attainment, social welfare, region of residence, marital status and alcohol use disorder

### Fatal breast cancer and DUD

In the study population, 0.4% (*n* = 16,959) died of breast cancer during the study period. We found a significant association between DUD and fatal breast cancer after adjustment for age in Model 1 (HR 1.63, 95% CI 1.44–1.84) and remained significant after adjustment also for age, educational attainment, social welfare, region of residence, marital status and alcohol use disorder in Model 2 (HR 1.60, 95% CI 1.42–1.82). In Model 2, fatal breast cancer was associated with living in a large city (HR 1.33, 95% CI 1.29–1.37), being not married/cohabiting (HR 1.35, 95% CI 1.31–1.39), of age 35–49 (HR 5.58, 95% CI 5.21–5.98) and 50–75 years (HR 14.44, 95% CI 13.53–15.42), and alcohol use disorder (HR 0.64, 95% 0.55–0.74). No statistically significant associations between fatal breast cancer and educational attainment or social welfare were found (Table 3).

**Table 3:** Association of drug use disorders (DUD) and fatal breast cancer

Covariates	Person years follow-up	Model 1				Model 2			
		HR	95% CI		*p*-value	HR	95% CI		*p*-value
DUD (vs. non)	862,294	1.63	1.44	1.84	< 0.001	1.60	1.42	1.82	< 0.001
Age (vs. age 15–34 years)									
35–49	17,729,577	5.14	4.80	5.50	< .0001	5.58	5.21	5.98	< .0001
50–75	22,030,892	13.17	12.37	14.02	< .0001	14.44	13.53	15.42	< .0001
Educational attainment < 12 years (vs. 12 + years)	35,461,860					0.99	0.96	1.03	0.6548
Social welfare (vs. no social welfare)	4,540,739					1.01	0.94	1.08	0.8158
Region of residence large city (vs. small city/countryside)	26,796,656					1.33	1.29	1.37	< .0001
Marital status not married/cohabiting (vs. married/cohabiting)	28,734,712					1.35	1.31	1.39	< .0001
Alcohol use disorder (vs. non)	1,039,030					0.64	0.55	0.74	< .0001

*N* = 3,838,248. Total person years follow-up 64,740,925

*HR* hazard ratio, *CI* confidence interval

Model 1: Adjusted for age; Model 2: Adjusted for age, educational attainment, social welfare, region of residence, marital status and alcohol use disorder

### Breast cancer stage and DUD

After exclusion of women without data on TNM-stage, 55,234 (50.7% of the 108,986 women with breast cancer) remained for the analysis. Of these, 673 had DUD. In the sub-sample of women with breast cancer, DUD was associated with stage II (HR 1.13, 95% CI 1.01–1.27) and stage IV (HR 2.06, 95% CI 1.44–2.95) after adjustment for age, social welfare, educational attainment, region of residence, marital status and alcohol use disorder (Table 4).

**Table 4:** Association of drug use disorders and breast cancer stage at time of diagnosis among women with malignant breast cancer

Cancer stage	No. of cases	HR*	95% CI		*p*-value
Stage 0	429	1.45	0.63	3.33	0.3811
Stage I	28,176	0.91	0.81	1.02	0.1130
Stage II	23,145	1.13	1.01	1.27	0.0402
Stage III	2016	1.26	0.88	1.81	0.2008
Stage IV	1468	2.06	1.44	2.95	< 0.001

*N* = 3,873,741. Cases of breast cancer = 55,234. Total person years follow-up 63,536,723

*HR* hazard ratio, *CI* confidence interval

*Full adjusted (for age, educational attainment, social welfare, region of residence, marital status and alcohol use disorder)

### Additional analyses

We conducted an additional analysis of incident and fatal breast cancer on a sub-sample of women with available information on tobacco smoking history, parity, age at first delivery, and BMI. DUD was significantly associated with fatal breast cancer (HR 1.63; 95% CI 1.21–2.19; *p* = 0.0013), when adjusting for age, educational attainment, social welfare, region of residence, marital status, alcohol use disorder, tobacco smoking, parity, age at first delivery, and BMI (Supplementary Table 2).

An additional analysis of incident and fatal breast cancer on a sub-sample of women with available information on family history of breast cancer showed that DUD was significantly associated with fatal breast cancer (HR 1.69; 95% CI 1.45–1.98; *p* < 0.001), when adjusting for age, educational attainment, social welfare, region of residence, marital status, alcohol use disorder, and family history of breast cancer (Supplementary Table 3).

We found no association between opioid use disorder and incident breast cancer or fatal breast cancer (Supplementary Table 4).

## Discussion

In this population-based study, we identified DUD as a predictor for incident breast cancer, fatal breast cancer, and metastasis at time of breast cancer diagnosis.

To our knowledge, this is the first large-scale study of breast cancer incidence, mortality and stage at diagnosis among women with DUD. Since DUD is frequently associated with alcohol and tobacco dependence [11, 19, 20, 23, 24], we have adjusted for these two risk factors of breast cancer [17, 18, 22]. In additional analyses that adjusted for risk factors for breast cancer [17, 18] (smoking, parity, age at first delivery and BMI; and family history of breast cancer), the association between DUD and incident breast cancer became statistically non-significant, suggesting that the risk factors listed above are likely to confound our results for incident but not for fatal breast cancer.

In addition to the above mentioned risk factors, the increased breast cancer mortality and incidence of metastatic breast cancer among women with DUD might be due to barriers towards seeking healthcare and attending breast cancer screening [12]. Previous studies have identified people who use drugs as having a high degree of unmet healthcare needs, barriers towards seeking healthcare and experience of stigma during healthcare encounters [9–11, 28]. Drug use is further often associated with poor SES, psychosocial vulnerability [8, 29] and psychiatric comorbidity [6, 7]; these factors are also associated with low attendance to screening with mammography [30, 31].

Our data on breast cancer mortality are incoherent with an Australian study by Randall et al. finding that opioid dependence was associated with *lower* breast cancer mortality compared to the general population [4]. The authors suggested that their finding may reflect the presence of protective factors for risk of breast cancer among women with DUD, such as giving birth at early age and having had multiple pregnancies. However, their study did not investigate breast cancer incidence. We conducted an additional analysis on opioid use disorder as the independent variable, and found no significant association with incident breast cancer or fatal breast cancer. As opioid dependence is associated with significantly increased mortality due to both drug-related (mainly overdose) and non-drug-related causes of death [1–3], it is possible that fatalities due to other causes than breast cancer might explain the results from Randall et al. [4].

In addition to our main results, we found that low educational attainment and receiving social welfare were inversely associated with breast cancer incidence but not mortality, which is in line with previous research identifying high SES as a risk factor for breast cancer [16]. Carlsen et al. [16] suggest that the positive association with SES may be explained by differences in total parity and age at first full-term pregnancy. Being not married/cohabiting and living in a large city were predictors of both incident and fatal breast cancer. Alcohol use disorder was associated with incident breast cancer but inversely associated with fatal breast cancer, which might be explained by high fatality rates related to excessive alcohol use, such as liver diseases and other serious diseases.

Our findings have important clinical implications. We have identified women with DUD as a risk group for incident, fatal and metastatic breast cancer, suggesting a need for raised awareness among health care professionals and decision-makers to improve breast cancer survival in women with DUD. Future research regarding cancer screening attendance, and potential barriers to healthcare seeking in general and cancer screening in particular, among women with DUD is needed to decrease these health inequities. We also suggest that assisted smoking cessation should be prioritized in treatment facilities for women with DUD.

## Strengths and limitations

This study analyzed nationwide data from several national registers of high quality. The Swedish Cancer Register has almost 100% validity and coverage [32–34], the Swedish Total Population Register is nearly 100% complete [35, 36], and the NPR for inpatient care is of documented high quality, with 85–95% of the diagnoses being valid [37].

Our definition of DUD as a composed variable from five national registers has been used in several previous studies [38, 39]. By using a broad definition ranging from a registration of DUD in the NPR to a registration of drug possession in the Suspicion Register, we aimed to include not only people with clinical DUD noted by the healthcare but also those who were suspected or convicted for a drug-related crime. On the other hand, we might have included women with a more sporadic drug use rather than a DUD. However, the 1.3% DUD from our register data is similar to the estimated 1.8% of the Swedish adult population who reported drug use, according to a survey made in 2017 by the Swedish Council for Information on Alcohol and Other Drugs [40].

Data on addiction treatment, types of substances used, or quantification of DUD, would have allowed for more refined analyses, but such data are not available on a total population level. Severe DUD, such as injection of heroin, is thought to be more stigmatizing and associated with lower health care seeking and more inadequate preventive health care compared to occasional, recreational drug use. On the other hand, a more severe DUD—especially involving opioids—might be associated with lower cancer mortality due to premature, drug-related death [1, 2]. This hypothesis is supported by our additional analysis and constitutes a subject for future research.

## Conclusion

In this study, we found that women who use drugs have higher incidence of breast cancer when compared to the general female population. We also found an association between DUD and fatal breast cancer as well as metastatic breast cancer when receiving the cancer diagnosis. Our findings should raise attention among medical staff and decision-makers towards a group of women in need of easily accessible breast cancer examinations.

## Electronic supplementary material

Below is the link to the electronic supplementary material.

Electronic supplementary material 1 (DOCX 246 kb)

## Data Availability

The data used to support the findings of this study are restricted by the Regional Ethical Review Board in Lund, Sweden, in order to protect patient privacy. Data are available from Kristina Sundquist, kristina.sundquist@med.lu.se for researchers who meet the criteria for access to confidential data.

## Abbreviations

BMI: Body mass index
DUD: Drug use disorders
NPR: National patient register
OST: Opioid substitution treatment
SES: Socioeconomic status
