# Supplementary material for: Drug use disorder and risk of incident and fatal breast cancer: a nationwide epidemiological study
Source: Breast Cancer Res Treat. 2020 Nov 6;186(1):199–207. doi: 10.1007/s10549-020-05998-4 (PMC7940313; doi:10.1007/s10549-020-05998-4)

**Supplementary Table 1. Population and number of events of drug use disorders (DUD). N=3,838,248.**

|  | | | | | | | | |
| --- | --- | --- | --- | --- | --- | --- | --- | --- |
|  | Without DUD | |  | With DUD | |  | Total population | |
|  | No | % |  | No | % |  | No | % |
| Age group (years) |  |  |  |  |  |  |  |  |
| 15-34 | 1418149 | 37.4 |  | 25220 | 49.6 |  | 1443369 | 37.6 |
| 35-49 | 998678 | 26.4 |  | 14838 | 29.2 |  | 1013516 | 26.4 |
| 50-75 | 1370563 | 36.2 |  | 10800 | 21.2 |  | 1381363 | 36.0 |
| Educational attainment |  |  |  |  |  |  |  |  |
| < 12 years | 2110535 | 55.7 |  | 33397 | 65.7 |  | 2143932 | 55.9 |
| 12 + years | 1676855 | 44.3 |  | 17461 | 34.3 |  | 1694316 | 44.1 |
| Social welfare |  |  |  |  |  |  |  |  |
| Non | 3534530 | 93.3 |  | 35153 | 69.1 |  | 3569683 | 93.0 |
| Yes | 252860 | 6.7 |  | 15705 | 30.9 |  | 268565 | 7.0 |
| Region of residence |  |  |  |  |  |  |  |  |
| Large city | 1567140 | 41.4 |  | 27365 | 53.8 |  | 1594505 | 41.5 |
| Small city/countryside | 2220250 | 58.6 |  | 23493 | 46.2 |  | 2243743 | 58.5 |
| Marital status |  |  |  |  |  |  |  |  |
| Married/cohabiting | 2115506 | 55.9 |  | 15246 | 30.0 |  | 2130752 | 55.5 |
| Not married/cohabiting | 1671884 | 44.1 |  | 35612 | 70.0 |  | 1707496 | 44.5 |
| Alcohol use disorder |  |  |  |  |  |  |  |  |
| Non | 3738554 | 98.7 |  | 36828 | 72.4 |  | 3775382 | 98.4 |
| Yes | 48836 | 1.3 |  | 14030 | 27.6 |  | 62866 | 1.6 |
| Incident breast cancer |  |  |  |  |  |  |  |  |
| Non | 3679714 | 97.2 |  | 49548 | 97.4 |  | 3729262 | 97.2 |
| Yes | 107676 | 2.8 |  | 1310 | 2.6 |  | 108986 | 2.8 |
| Fatal breast cancer |  |  |  |  |  |  |  |  |
| Non | 3770698 | 99.6 |  | 50591 | 99.5 |  | 3821289 | 99.6 |
| Yes | 16692 | 0.4 |  | 267 | 0.5 |  | 16959 | 0.4 |
| All | 3787390 | 100.0 |  | 50858 | 100.0 |  | 3838248 | 100.0 |

**Supplementary Table 2. Association of drug use disorders (DUD) and incident breast cancer and fatal breast cancer, with additional adjustment for tobacco smoking history, parity, age at first delivery and**

|  | | | | | | | | | | | | |
| --- | --- | --- | --- | --- | --- | --- | --- | --- | --- | --- | --- | --- |
|  | Incident breast cancer | | | | | | Fatal breast cancer | | | | | |
| Total number of cases | 22160 | | | | | | 2239 | | | | | |
| Total person years follow-up | 23759714 | | | | | | 23906718 | | | | | |
| Covariates | No. of cases | Person years follow-up | HR* | 95% CI | | P-value | No. of cases | Person years follow-up | HR* | 95% CI | | P-value |
| DUD (vs. non) | 296 | 388390 | 0.95 | 0.84 | 1.07 | 0.3992 | 49 | 390335 | 1.63 | 1.21 | 2.19 | 0.0013 |
| Age (vs. age 15-34 years) |  |  |  |  |  |  |  |  |  |  |  |  |
| 35-49 | 12613 | 6596052 | 3.45 | 3.34 | 3.55 | <.0001 | 1340 | 6692299 | 4.19 | 3.80 | 4.63 | <.0001 |
| 50-75 | 1095 | 305063 | 5.79 | 5.40 | 6.21 | <.0001 | 164 | 314103 | 9.42 | 7.77 | 11.42 | <.0001 |
| Educational attainment <12 years (vs. 12+ years) | 8077 | 7740338 | 0.95 | 0.93 | 0.98 | <.0001 | 945 | 7794996 | 1.13 | 1.04 | 1.24 | 0.0061 |
| Social welfare (vs. no social welfare) | 1634 | 2475289 | 0.83 | 0.79 | 0.87 | <.0001 | 215 | 2485115 | 1.06 | 0.91 | 1.22 | 0.4503 |
| Region of residence large city (vs. small city/countryside) | 11524 | 11001645 | 1.19 | 1.16 | 1.23 | <.0001 | 1155 | 11078169 | 1.17 | 1.08 | 1.27 | 0.0002 |
| Marital status not married/cohabiting (vs. married/cohabiting) | 9755 | 14014844 | 0.82 | 0.79 | 0.84 | <.0001 | 985 | 14074149 | 0.88 | 0.80 | 0.96 | 0.0045 |
| Alcohol use disorder (vs. non) | 402 | 420604 | 1.01 | 0.92 | 1.12 | 0.7991 | 28 | 423313 | 0.54 | 0.36 | 0.79 | 0.0016 |
| Smoking history (vs. non smoking) | 4776 | 3821975 | 1.19 | 1.15 | 1.23 | <.0001 | 526 | 3854875 | 1.16 | 1.05 | 1.29 | 0.0045 |
| Parity (vs. one child) |  |  |  |  |  |  |  |  |  |  |  |  |
| Two children | 11070 | 11725234 | 1.05 | 1.01 | 1.08 | 0.0111 | 1041 | 11797921 | 0.90 | 0.81 | 1.00 | 0.055 |
| Three children | 4673 | 4964150 | 0.95 | 0.91 | 0.99 | 0.0157 | 463 | 4995123 | 0.85 | 0.75 | 0.97 | 0.0119 |
| Four or more children | 1282 | 1679612 | 0.75 | 0.71 | 0.80 | <.0001 | 145 | 1688205 | 0.73 | 0.61 | 0.88 | 0.0008 |
| Age at first delivery (vs. age <30 years) |  |  |  |  |  |  |  |  |  |  |  |  |
| 30-39 | 12323 | 11776880 | 1.16 | 1.12 | 1.19 | <.0001 | 1237 | 11860824 | 1.13 | 1.03 | 1.24 | 0.0098 |
| 40+ | 1254 | 753811 | 1.32 | 1.24 | 1.41 | <.0001 | 154 | 763012 | 1.32 | 1.09 | 1.60 | 0.0047 |
| BMI (vs. 18.5-24.9) | 10539 | 12622957 | 0.98 | 0.95 | 1.00 | 0.0586 | 1110 | 12700455 | 1.06 | 0.98 | 1.16 | 0.1519 |

**body mass index (BMI). N=1,349,971.**

HR=Hazard ratio. CI=confidence interval.

*: Full adjusted (for age, educational attainment, social welfare, region of residence, marital status, alcohol use disorder, smoking history, parity, age at first delivery and BMI).

**Supplementary Table 3. Association of drug use disorders (DUD) and incident breast cancer and fatal breast cancer, with additional adjustment for family history of breast cancer. N=2,458,279.**

|  | | | | | | | | | | | | |
| --- | --- | --- | --- | --- | --- | --- | --- | --- | --- | --- | --- | --- |
|  | Incident breast cancer | | | | | | Fatal breast cancer | | | | | |
| Total number of cases | 73003 | | | | | | 9168 | | | | | |
| Total person years follow-up | 42303655 | | | | | | 42876370 | | | | | |
| Covariates | No. of cases | Person years follow-up | HR* | 95% CI | | P-value | No. of cases | Person years follow-up | HR* | 95% CI | | P-value |
| DUD (vs. non) | 911 | 670440 | 1.02 | 0.96 | 1.09 | 0.5340 | 173 | 677631 | 1.69 | 1.45 | 1.98 | <.0001 |
| Age (vs. age 15-34 years) |  |  |  |  |  |  |  |  |  |  |  |  |
| 35-49 | 27697 | 13229668 | 4.29 | 4.19 | 4.40 | <.0001 | 3236 | 13449339 | 5.40 | 4.99 | 5.83 | <.0001 |
| 50-75 | 36090 | 10194807 | 7.43 | 7.25 | 7.62 | <.0001 | 5065 | 10501617 | 10.94 | 10.13 | 11.81 | <.0001 |
| Educational attainment <12 years (vs. 12+ years) | 39587 | 19331017 | 0.88 | 0.87 | 0.90 | <.0001 | 5498 | 19645149 | 1.06 | 1.01 | 1.11 | 0.0114 |
| Social welfare (vs. no social welfare) | 2960 | 2932310 | 0.86 | 0.83 | 0.89 | <.0001 | 432 | 2952950 | 1.02 | 0.92 | 1.12 | 0.7448 |
| Region of residence large city (vs. small city/countryside) | 36614 | 19596700 | 1.18 | 1.17 | 1.20 | <.0001 | 4608 | 19881309 | 1.20 | 1.15 | 1.25 | <.0001 |
| Marital status not married/cohabiting (vs. married/cohabiting) | 29519 | 23057359 | 1.00 | 0.98 | 1.01 | 0.6677 | 3879 | 23275662 | 1.16 | 1.11 | 1.21 | <.0001 |
| Alcohol use disorder (vs. non) | 1406 | 826437 | 1.05 | 1.00 | 1.11 | 0.0597 | 121 | 836770 | 0.61 | 0.51 | 0.73 | <.0001 |
| Family history of breast cancer (vs. non) | 12309 | 3799566 | 1.78 | 1.75 | 1.82 | <.0001 | 1518 | 3899907 | 1.70 | 1.61 | 1.79 | <.0001 |

HR=Hazard ratio. CI=confidence interval.

*: Full adjusted (for age, educational attainment, social welfare, region of residence, marital status, alcohol use disorder and family history of breast cancer).

**Supplementary Table 4. Association of opioid use disorder (ICD-10 F11) and incident breast**

|  | | | | | | | | | | | | |
| --- | --- | --- | --- | --- | --- | --- | --- | --- | --- | --- | --- | --- |
|  | Incident breast cancer | | | | | | Fatal breast cancer | | | | | |
| Total number of cases | 108986 | | | | | | 16959 | | | | | |
| Total person years follow-up | 63903192 | | | | | | 64740925 | | | | | |
| Covariates | No. of cases | Person years follow-up | HR* | 95% CI | | P-value | No. of cases | Person years follow-up | HR* | 95% CI | | P-value |
| Opioid use disorders (ICD-10 F11)** (vs. non) | 151 | 106474 | 0.99 | 0.84 | 1.16 | 0.8708 | 29 | 107668 | 1.35 | 0.94 | 1.94 | 0.1074 |
| Age (vs. age 15-34 years) |  |  |  |  |  |  |  |  |  |  |  |  |
| 35-49 | 32798 | 17472852 | 4.58 | 4.48 | 4.68 | <.0001 | 3868 | 17729577 | 5.57 | 5.20 | 5.97 | <.0001 |
| 50-75 | 65056 | 21504144 | 7.95 | 7.79 | 8.13 | <.0001 | 12030 | 22030892 | 14.39 | 13.47 | 15.36 | <.0001 |
| Educational attainment <12 years (vs. 12+ years) | 65686 | 34957420 | 0.77 | 0.76 | 0.78 | <.0001 | 11652 | 35461860 | 0.99 | 0.96 | 1.03 | 0.7451 |
| Social welfare (vs. no social welfare) | 5225 | 4504849 | 0.89 | 0.87 | 0.92 | <.0001 | 835 | 4540739 | 1.02 | 0.95 | 1.10 | 0.5295 |
| Region of residence large city (vs. small city/countryside) | 54934 | 26375130 | 1.36 | 1.34 | 1.37 | <.0001 | 8432 | 26796656 | 1.33 | 1.29 | 1.37 | <.0001 |
| Marital status not married/cohabiting (vs. married/cohabiting) | 43639 | 28414388 | 1.14 | 1.13 | 1.15 | <.0001 | 7174 | 28734712 | 1.36 | 1.31 | 1.40 | <.0001 |
| Alcohol use disorder (vs. non) | 1885 | 1025119 | 1.13 | 1.08 | 1.18 | <.0001 | 182 | 1039030 | 0.69 | 0.60 | 0.80 | <.0001 |

**cancer and fatal breast cancer. N=3,838,248.**

HR=Hazard ratio. CI=confidence interval.

*: Full adjusted (for age, educational attainment, social welfare, region of residence, marital status and alcohol use disorder).

**: N=6,400.

**Figure legends:**

**Supplementary Figure 1. Flow chart documenting inclusion criteria, exclusion criteria, and the number of individuals included in the study population.**

**Supplementary Figure 2. Plot to assess the proportional hazard assumption of categorical predictor DUD using Schoenfeld Residuals.**

**
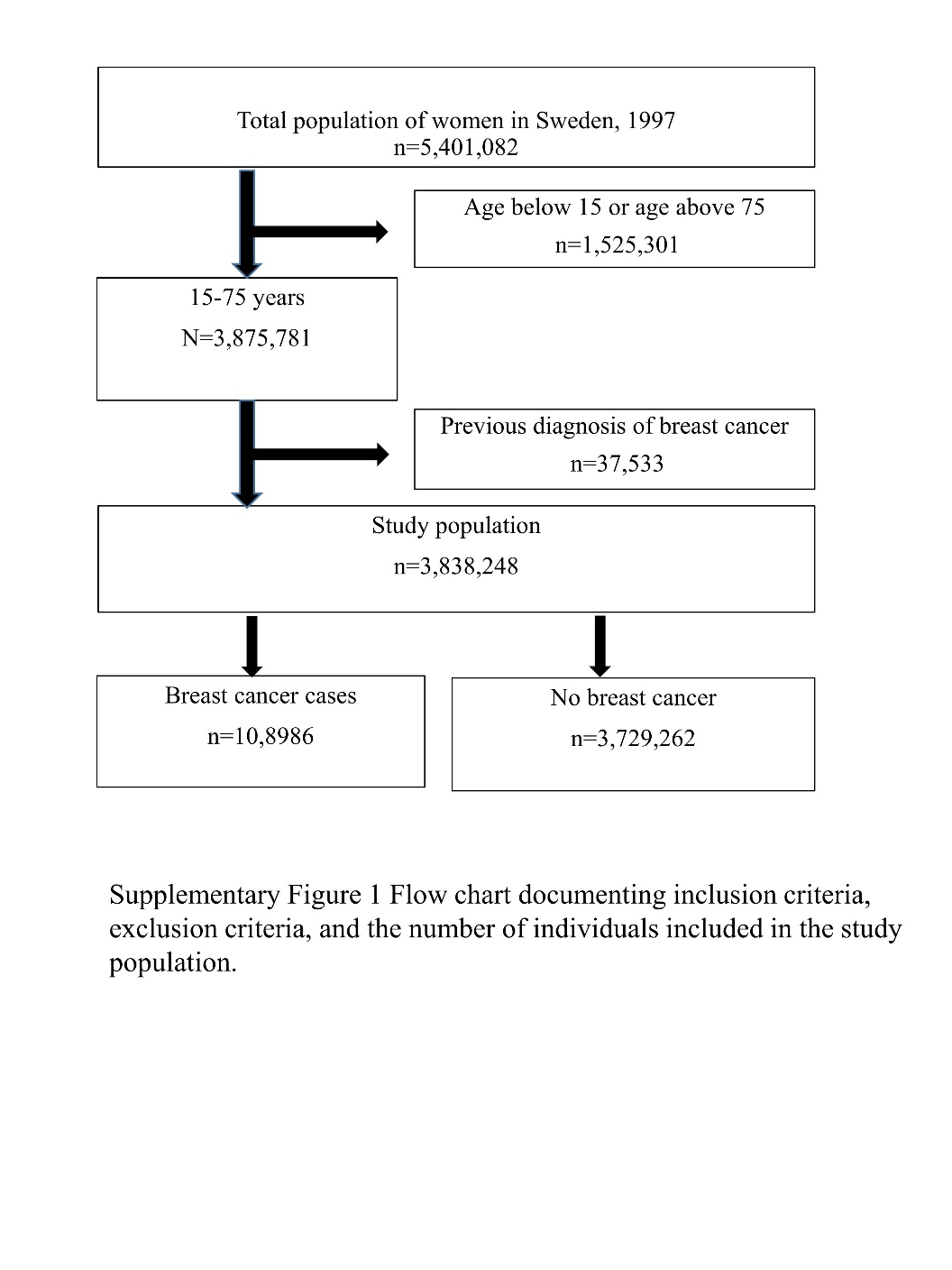
**

**Supplementary Figure 2. Plot to assess the proportional hazard assumption of categorical predictor DUD using Schoenfeld Residuals**


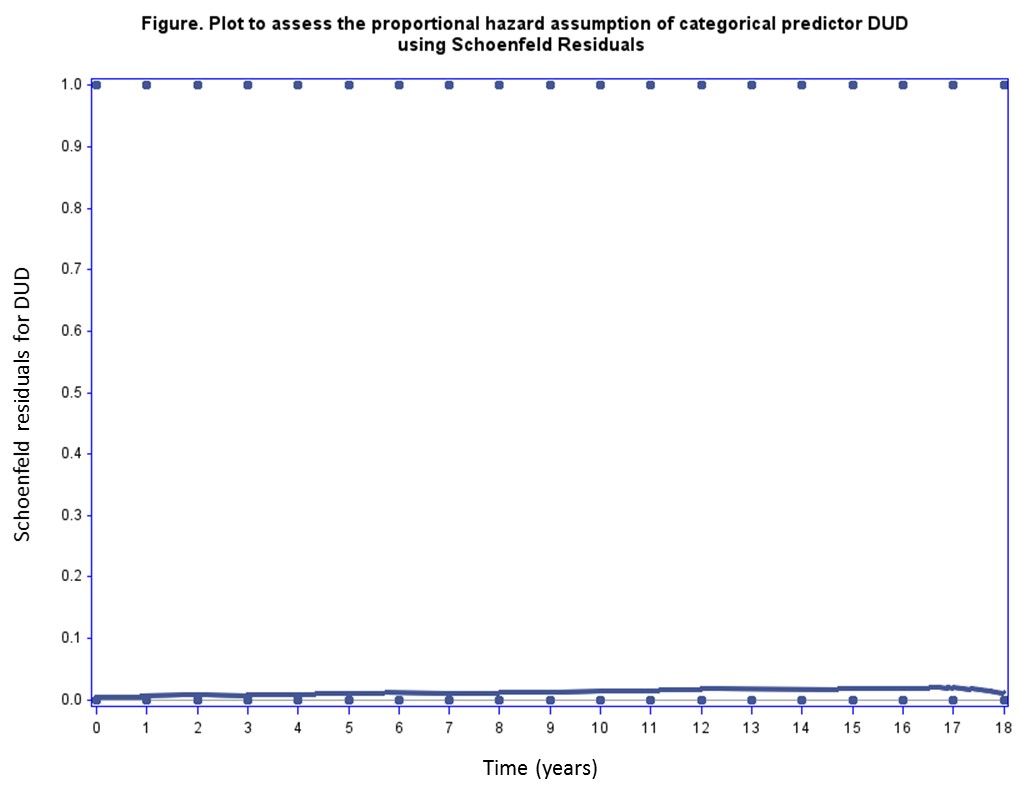

Supplement: Supplementary file 1 — Electronic supplementary material 1 (DOCX 246 kb) [file 10549_2020_5998_MOESM1_ESM.docx]
